# Supplementary material for: Identification of Human Breast Adipose Tissue Progenitors Displaying Distinct Differentiation Potentials and Interactions with Cancer Cells
Source: Biomedicines. 2022 Aug 9;10(8):1928. doi: 10.3390/biomedicines10081928 (PMC9406003; doi:10.3390/biomedicines10081928)
Supplement: Supplementary file 1 [file biomedicines-10-01928-s001.zip › biomedicines-1789373-supplementary.pdf]

Supplementary Information

Identification of Human Breast Adipose Progenitors Displaying Distinct Differentiation Potentials and Interactions with Cancer Cells

Pascal PERALDI[1], Agnès LOUBAT[1], Bérengère CHIGNON-SICARD[1-2], Christian DANI[1], Annie LADOUX[1]

Material and Methods

Reagents

Sequences for the primers used in this study

Gene	Forward primer	Reverse primer
INHBA	5’- GGGAGAACGGGTATGTGGAGAT-3’	5’-GCTGTTCCTGACTCGGCAAA-3’
ALPL (MSCA1)	5’- GCGCAAGAGACACTGAAATATGC-3’	5’- TGGTGGAGCTGACCCTTGAG-3’
*CD142**	5’- GGAACCCAAACCCGTCAATC-3’	5’-GTGCCAAGTACGTCTGCTTC-3’
TNFα	5’-TCTTCTCGAACCCCGAGTGA-3’	5’-CCTCTGATGGCACCACCAG-3’
IL1β	5’-ACAGATGAAGTGCTCCTTCCA-3’	5’- GTCGGAGATTCGTAGCTGGAT-3’
TGF-βI	5’-GGAAACCCACAACGAAATCTATGA-3’	5’-GAGAGCAACACGGGTTCAGGTA -3’
TGFβRI	5’- CCCTCTTCAAAAACTGGGTCTGT-3’	5’- AAGCACACTGGTCCAGCAATG -3’
TNF-R1	5’-GATACGGACTGCAGGGAGTG-3’	5’- CTGAGGCAGTGTCTGAGGTG-3’
IL1-R	5’-AGGGATGACTACGTTGGGGA-3’	5’- TTCAGATGAACCACCCAGCC-3’
36B4	5'-CTACAACCCTGAAGAAGTGCTTG-3’	5’- CAATCTGCAGACAGACACTGG-3’
CD34	5’-CCCAGCCAACGTTTCAACTC-3’	5’-GCAAGGCTAGTGCTAGTGGT-5’

(*These primers can amplify the two isoforms of human *CD142*)

Antibodies

Antibodies used for FACS analysis

ANTIBODY	MANUFACTURER	CATALOG NUMBER	Fluorochrome
ANTI-CD73	Miltenyi Biotec	130-095-182	Phycoerythrin
ANTI-CD90	BD Pharmingen	555596	Phycoerythrin
ANTI-CD105	R&D Systems	FAB 10971F	Fluorescein
ANTI-CD142	Invitrogen eBioscience	12-1429-42	Phycoerythrin

Antibodies used for Western Blots and immunohistochemistry analysis

ANTIBODY	MANUFACTURER	CATALOG NUMBER	DILUTION
ANTI-PLIN1	ACRIS Gmbh	#BP5015	10000 (WB) 1000 (ICC)
ANTI-Tubulin β1	SIGMA ALDRICH	#T7816	10000 (WB)
ANTI-alpha SMA	SIGMA ALDRICH	#A5228	2000(WB) 1000 (ICC)
ANTI-N-Cadherin	GeneTex	#GTX 127345	1000 (WB)
ANTI-UCP1	Millipore	#662045	1000 (WB)
ANTI- CD274 (PD-L1)	GeneTex	# GTX104763	1000 (WB) 600 (ICC)
ANTI-Acetylated tubulin (clone 6-11B-1)	SIGMA ALDRICH	#T7451	5000
ANTI-Pericentrin	EUROMEDEX	#A301-348A	5000
Anti Estrogen receptor-α	GeneTex	#GTX127978	1000
Anti Estrogen receptor-β	ABCAM	#3576	1000

Methods

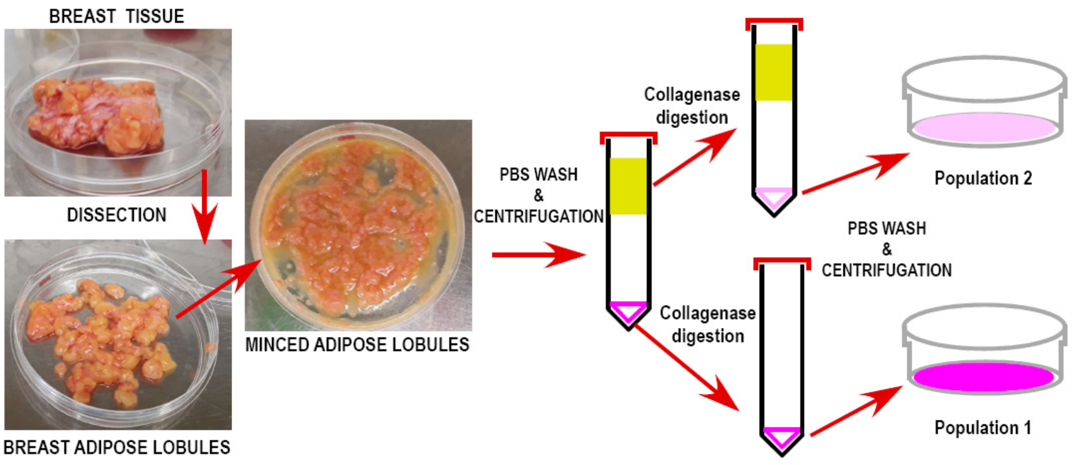

Supplementary Figure S1: Schematic representation of the protocol used to isolate Adipose Progenitor Cells from human breast.

Breast APCs were derived from the stroma vascular fraction of women who underwent breast reduction procedures. Adipose tissue was dissected to obtain adipose lobules that were minced. A first centrifugation step allowed separation of the fat and lean fractions. Digestion of both fractions was performed with collagenase A (10mg/ml) in PBS/BSA (2% w/v) for 30 min at 37°C under gentle stirring. Cells of the stroma vascular fraction (SVF) were collected by centrifugation at 260g for 5 min and further washed with PBS before plating.

Results

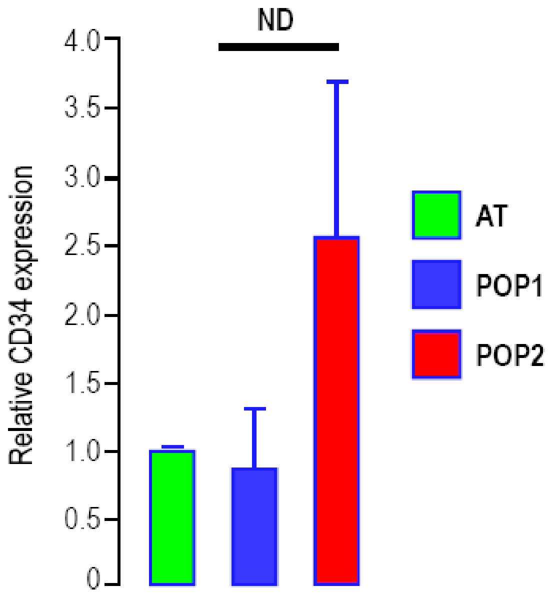

Supplementary Figure S2. Analysis of CD34 expression in the two APC populations. Expression of *CD34* was assessed by real-time RT-PCR and normalized for the expression of *36B4* mRNA for all the patients. cDNA from human adipose tissue (AT) was used as a positive control. The means ± SEM were calculated from 2 to 4 independent experiments, with determinations performed in duplicate for each patient (ND: not different).

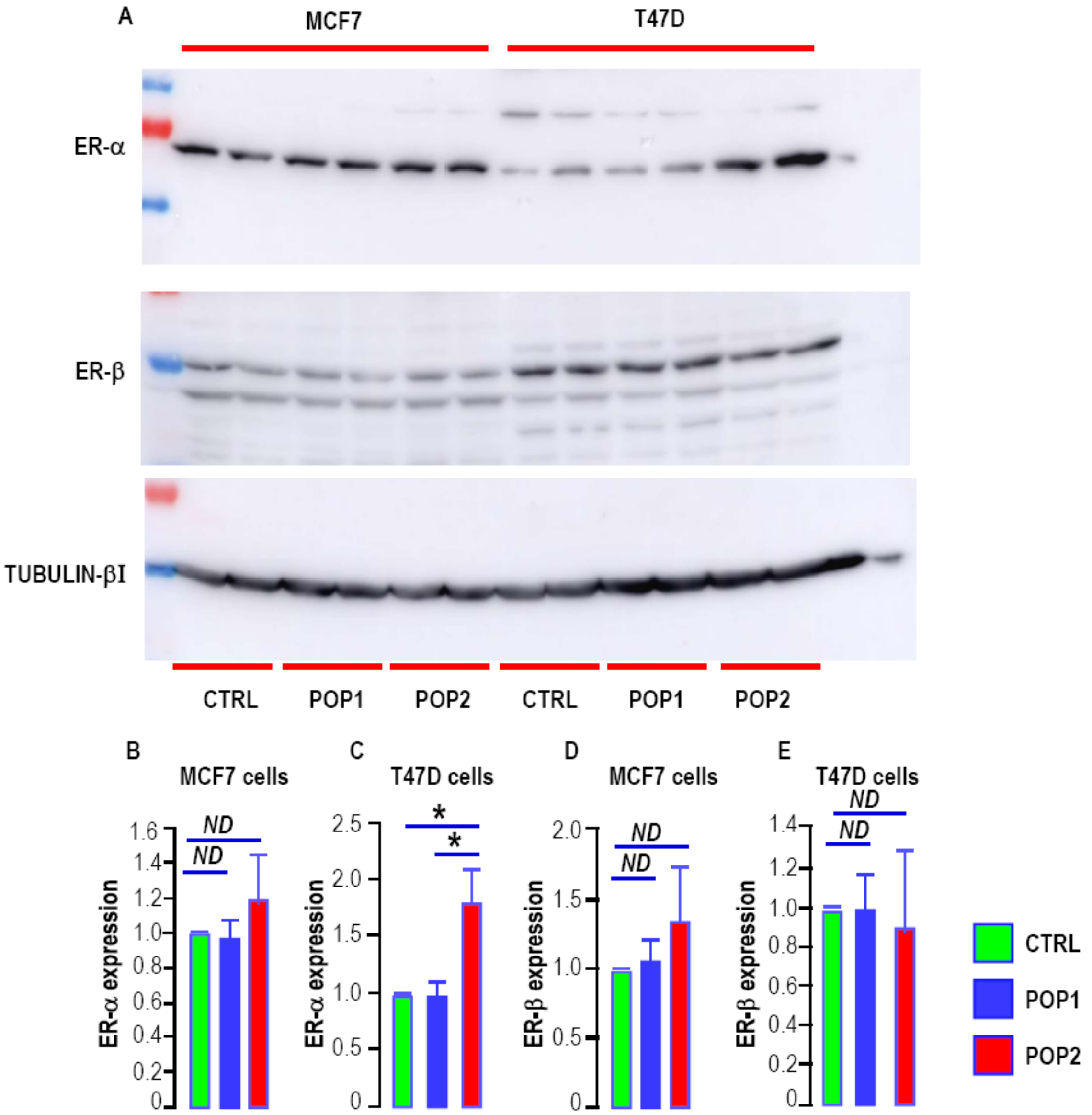

Supplementary Figure S3. Expression of estrogen receptors in MCF7 and T47D cells co-cultured with the APC populations. A) Protein expression was measured in the two breast cancer cell lines that have been co-incubated, or not (control: CTRL), in presence of APC populations 1 (POP1) or 2 (POP2). Expressions of ER-α or ER-β were measured by SDS PAGE using 10% acrylamide gels. Tubulin-βI was used as a loading control. Expressions of the two proteins were analysed by Western Blots using specific antibodies. Representative Western blots are shown. **B-E)** Quantification of the signals. Protein expression was quantified using FIJI program and compared to the expression of Tubulin-βI. The means + SEM were calculated from five independent experiments (*p<0.05; ND: not different)

Supplementary Table S1. Patient information.

PATIENT ID	AGE (years)	BMI
1	44	26,6
2	50	22,04
3	25	21,8
4	40	25,6
5	28	24,1

Table S1 recapitulates the age and the body mass index of the patients.

Supplementary Table S2. Time lapse analysis.

PATIENT	MEAN	n= 175		MEAN	n=263
1	2,104	23		1,31	9
	2,59	24		1,708	11
				2,13	27
				2,21	43
2	2,26	20		1,92	24
	2,305	27		1,77	15
3	2,129	46		2,065	45
4	2,09	27		2,1242	20
	2,149	18		1,84	18
5	2,149	30		1,69	51
**MEAN**	**2,222**			**1,877**	
**SE**	**0,059**			**0,086**	
**p=**	**0,0029**				

This table recapitulates the time lapse measurements for each patient. The mean area covered by the MCF7 mammospheres and the number of events analyzed for each experiments are indicated.

Supplementary Videos 1 and 2

Adipose progenitor cells (APCs) from population1 (Video1) or population 2 (video 2) were grown and differentiated on glass coverslips mounted in a specific adaptor for microscopy. Breast mammospheres expressing GFP were added 24 hour prior the beginning of the recording, for them to adhere to the APCs grown on coverslips. The time lapse experiments were performed on an inverted AxioObserver - Zeiss microscope equipped with a sCMOS ANDOR Neo camera at 37°C in a CO_2-controlled atmosphere (Magnification 10X). The images were acquired every 20 min with total imaging time of 63 h. The system was controlled using MetaMorph software (Molecular Devices, Sunnyvale, CA). The assembly of the 190 images recorded for each channel (GFP or Nomarski) was performed with MetaMorph software.

Assembly of the images of the two channels was performed using Fiji. Typical recordings with APCs from patient 1 are shown.
